# Factors affecting dental biofilm in patients wearing fixed orthodontic appliances

**DOI:** 10.1186/s40510-016-0158-5

**Published:** 2017-01-30

**Authors:** Li Mei, Joyce Chieng, Connie Wong, Gareth Benic, Mauro Farella

**Affiliations:** 1 Discipline of Orthodontics, Department of Oral Sciences, Sir John Walsh Research Institute, Faculty of Dentistry, University of Otago, Dunedin, New Zealand; 2 Sir John Walsh Research Institute, Faculty of Dentistry, University of Otago, Dunedin, New Zealand

**Keywords:** Biofilm, White spot lesions, Oral hygiene, Orthodontics, Dental plaque

## Abstract

**Background:**

The aim of this study is to investigate the amount and the distribution of biofilm in patients wearing fixed appliances and its relation with age, gender, frequency of tooth brushing, and patient motivation.

**Methods:**

The sample comprised 52 patients (15.5 ± 3.6 years old, 30 females and 22 males) wearing fixed orthodontic appliances. Dental biofilm was assessed using a modified plaque index (PI). A questionnaire was used to collect patient’s information, including gender, age, treatment motivation, and frequency of tooth brushing.

**Results:**

Gingival (PI score = 0.9 ± 0.7), mesial (0.8 ± 0.6), and distal (0.8 ± 0.5) areas accumulated more biofilm than occlusal areas (0.3 ± 0.3) (*P* < 0.038). The maxillary lateral incisors (1.1 ± 0.8) and maxillary canines (1.0 ± 0.8) had more biofilm than other teeth (*P* < 0.05). The maxillary arch (0.8 ± 0.7) had significantly more biofilm than mandibular arch (0.6 ± 0.6) (*P* = 0.042). No significant difference was found between the right side (0.7 ± 0.7) and left side (0.7 ± 0.6) (*P* = 0.627). Less biofilm was found in females (0.6 ± 0.5), adults (0.3 ± 0.3), and “self-motivated” patients (0.3 ± 0.3), compared with males (0.9 ± 0.5), children (0.8 ± 0.6), and “family-motivated” patients (1.1 ± 0.5) (*P* < 0.001). The amount of biofilm was associated with self-report of the frequency of daily tooth brushing (*P* < 0.001).

**Conclusions:**

Patients wearing fixed orthodontic appliances have the highest biofilm accumulation on the maxillary lateral incisors and maxillary canines, particularly in the gingival area and areas behind arch wires. Less biofilm was observed in female and adult patients and in those who were self-motivated and brushed their teeth more often.

## Background

Biofilm formation around fixed orthodontic appliances can cause important side effects. This includes white spot lesions (WSLs) and, in severe cases, tooth decay, with a negative impact on patient’s quality of life [1, 2]. Although many auxiliary dental products such as interdental brushes, specialized toothbrushes, and mouth rinses are commercially available, the prevalence of WSLs still remains as high as 72.9% [3]. This is because the placement of fixed orthodontic appliances severely impedes tooth brushing, makes conventional oral hygiene procedures more difficult, and provides areas of low salivary flow that allow bacterial adhesion and biofilm formation [4, 5].

The introduction of fixed appliances into the oral cavity not only promotes the amount of biofilm formation but also increases the level of acidogenic bacteria inside the biofilm, resulting in a higher cariogenic challenge around orthodontic brackets and bands [6–8]. If patients cannot maintain good oral hygiene during orthodontic treatment, the acid produced by dental biofilms will eventually lead to enamel demineralization and WSLs. Though some superficial soft WSLs can be remineralized, most will persist after the removal of the fixed appliances [4].

The distribution of dental biofilm seen in patients wearing fixed orthodontic appliances may, with time, reflect the eventual distribution of WSLs [2, 9]. It has been confirmed that the presence of biofilm on the tooth surface is a predictive factor for the development of carious lesions in children [10]. The distribution of biofilm is significantly related to the distribution of gingivitis, and the greater the accumulation of biofilm, the higher the gingival bleeding index [9, 11]. For better WSL prevention in orthodontics, it is important to understand which factors influence the amount of dental biofilm and its distribution pattern in patients with fixed appliances.

The aims of this study are to investigate the amount and distribution of dental biofilm in patients wearing fixed appliances and to evaluate its association with age, gender, motivation to treatment, and frequency of tooth brushing.

## Methods

### Study design and participants

The study was designed as a cross-sectional study. A convenience sample of 127 orthodontic patients were screened at the Department of Orthodontics of the University of Otago, and 52 eligible patients (30 females and 22 males, mean age = 15.5 ± 3.6 years) were finally included in the study on the basis of sample size used in previous published researches [12, 13]. The patients were selected according to the following inclusion criteria: wearing fixed orthodontic appliances, at least 20 natural teeth, and a willingness to participate in the study; and to the following exclusion criteria: wearing lingual fixed appliances, extensive dental restorations, active periodontal disease, systemic diseases, or the use of medication which may influence periodontal health. This study was approved by the Human Ethics Committee of the University of Otago (13/106).

### Dental biofilm assessment

**Fig. 1:**
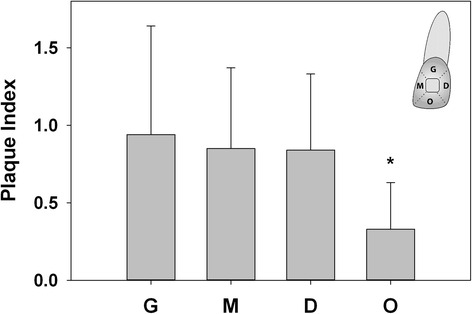
Biofilm formation on the four areas of a tooth in relation to the bracket (*G* gingival, *M* mesial, *D* distal, *O* occlusal). The occlusal area accumulated the least amount of biofilm compared with the gingival, mesial, and distal areas (*P* < 0.038). No significant difference was found among the gingival, mesial, and distal areas (*P* > 0.132)

All consenting patients were examined for the status of biofilm formation using the modified Silness and Löe plaque index (PI) and a periodontal probe [14]. Each tooth was divided into four areas in relation to the bracket: G = gingival; M = mesial; D = distal; and O = occlusal (Fig. 1). Plaque was then scored in each area based on the original Silness and Löe plaque index [14, 15]. The PI scores for all teeth except the second and third molars were measured and recorded [16]. All measurements were taken by two calibrated dental investigators (J.C. and C.W.). The agreement between examiners was assessed using kappa statistics (Kappa value = 0.84).

A questionnaire was used to collect patient information. This included gender, age, motivation to undergo orthodontic treatment (self-motivated, family-motivated, self- and family-motivated), and self-reported frequency of tooth brushing (times per day).

#### Statistical analysis

PI scores were reported as means, standard deviations, and 95% confidence intervals (95% CI) [12]. The data were analyzed using a mixed model analysis. The plaque index was considered as the response variable, whereas age, gender, motivation, frequency of tooth brushing, and site were entered into the models as covariates (fixed effect). A random term for study participant was also entered. The significance level was set at *P* < 0.05. All analyses were performed using SPSS software (version 17.0 for Windows; SPSS, Chicago, IL).

## Results

Biofilm formation on the four areas of a tooth in relation to the bracket was significantly different (*P* < 0.001). The gingival (PI score = 0.9 ± 0.7), mesial (0.8 ± 0.6), and distal (0.8 ± 0.5) areas accumulated greater amount of biofilm than the occlusal area (0.3 ± 0.3) (*P* ≤ 0.038) (Fig. 1). No significant difference was found among the gingival, mesial, and distal areas (*P* ≥ 0.132).

**Fig. 2:**
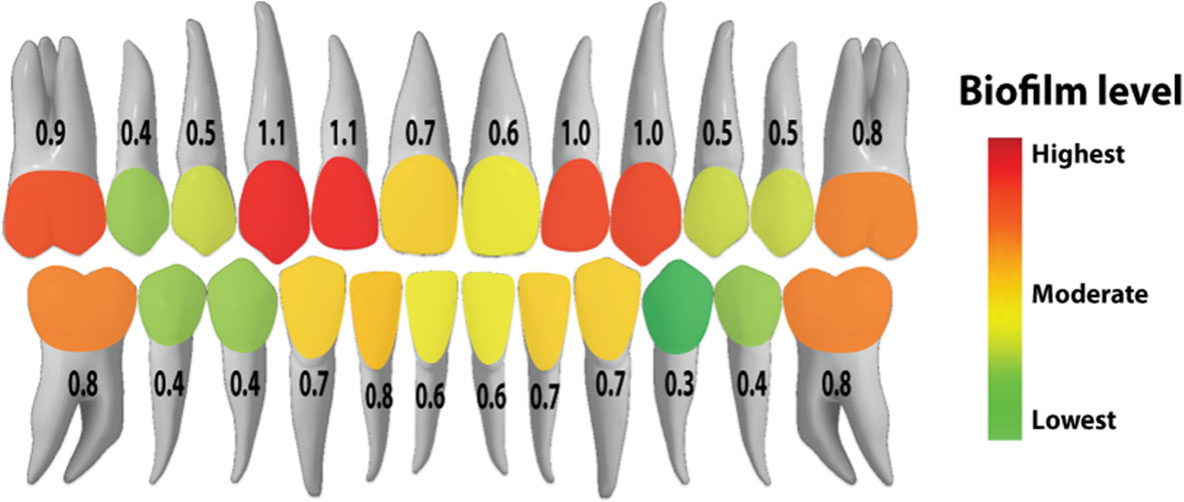
Mean levels of biofilm formation on each tooth as indicated by the plaque index and a color-coded map

Biofilm formation on each tooth is summarized in Table 1 and Fig. 2. The highest level of biofilm formation was found on the maxillary lateral incisors and maxillary canines, amounting to 1.1 ± 0.8 and 1.0 ± 0.8, respectively (*P* ≤ 0.043). These areas accumulated almost three times more biofilm than the mandibular premolars (0.4 ± 0.3), which had the lowest level of biofilm accumulation (*P* = 0.036) (Fig. 2).

**Table 1:** Plaque index (PI) score of each tooth

Quadrant	Tooth	Plaque index^a^	95% CI for mean	
			Lower bound	Upper bound
1	11	0.7	0.5	0.8
	12	1.1	0.8	1.3
	13	1.1	0.9	1.3
	14	0.5	0.4	0.7
	15	0.4	0.3	0.6
	16	0.9	0.7	1.1
Overall		0.8	0.6	1.0
2	21	0.6	0.4	0.8
	22	1.0	0.8	1.2
	23	1.0	0.8	1.2
	24	0.5	0.3	0.6
	25	0.5	0.3	0.7
	26	0.8	0.6	1.1
Overall		0.7	0.5	1.0
3	31	0.6	0.4	0.8
	32	0.7	0.5	1.0
	33	0.7	0.5	1.0
	34	0.3	0.2	0.5
	35	0.4	0.2	0.5
	36	0.8	0.6	1.0
Overall		0.6	0.4	0.8
4	41	0.6	0.4	0.8
	42	0.8	0.6	1.0
	43	0.7	0.5	1.0
	44	0.4	0.2	0.5
	45	0.4	0.2	0.5
	46	0.8	0.6	1.0
Overall		0.6	0.4	0.8

^a^Data represent mean

**Fig. 3:**
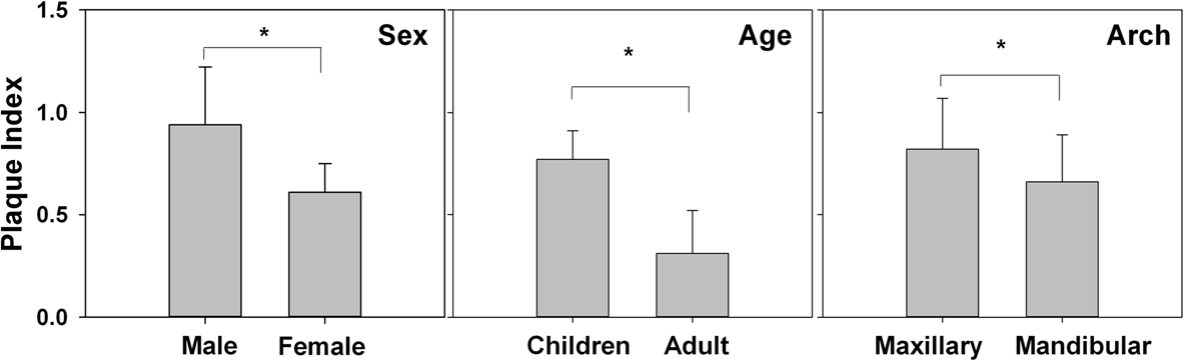
Comparison of biofilm formation by sex, age group, and arch. Males had significantly more biofilm than females (*P* < 0.001). Children had significantly more biofilm than adults (*P* < 0.001). The maxillary arch had significantly more biofilm than the mandibular arch (*P* = 0.042)

The maxillary arch (0.8 ± 0.7) had significantly more dental biofilm than the mandibular arch (0.6 ± 0.6) (*P* = 0.042), whereas no significant difference was found between the right side (0.7 ± 0.7) and left side (0.7 ± 0.6) (*P* = 0.627) (Fig. 3). The amount of biofilm formation did not differ significantly between quadrants (*P* ≥ 0.057) (Table 1).

**Fig. 4:**
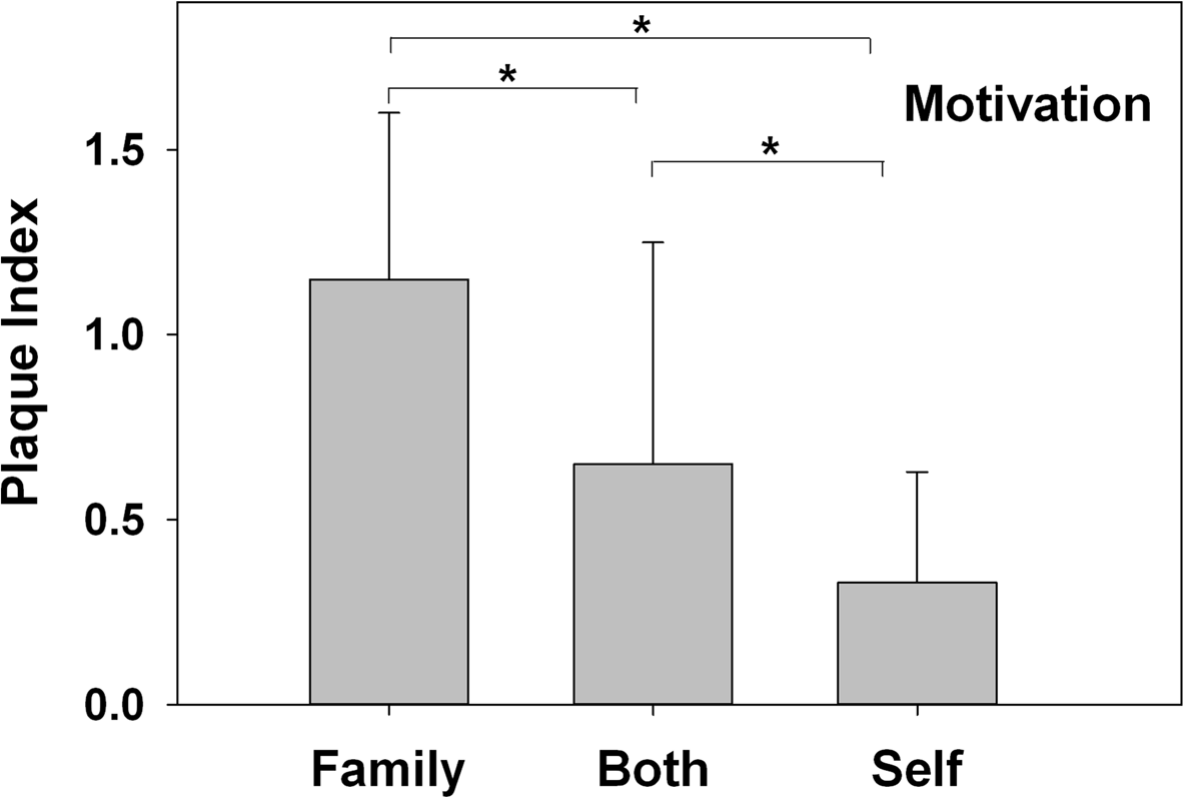
Biofilm formation in patients with different motivations to undergo orthodontic treatment. “Family-motivated” patients had more biofilm formation, followed by subjects who were “self- and family-motivated,” and “self-motivated” (*P* < 0.001)

**Fig. 5:**
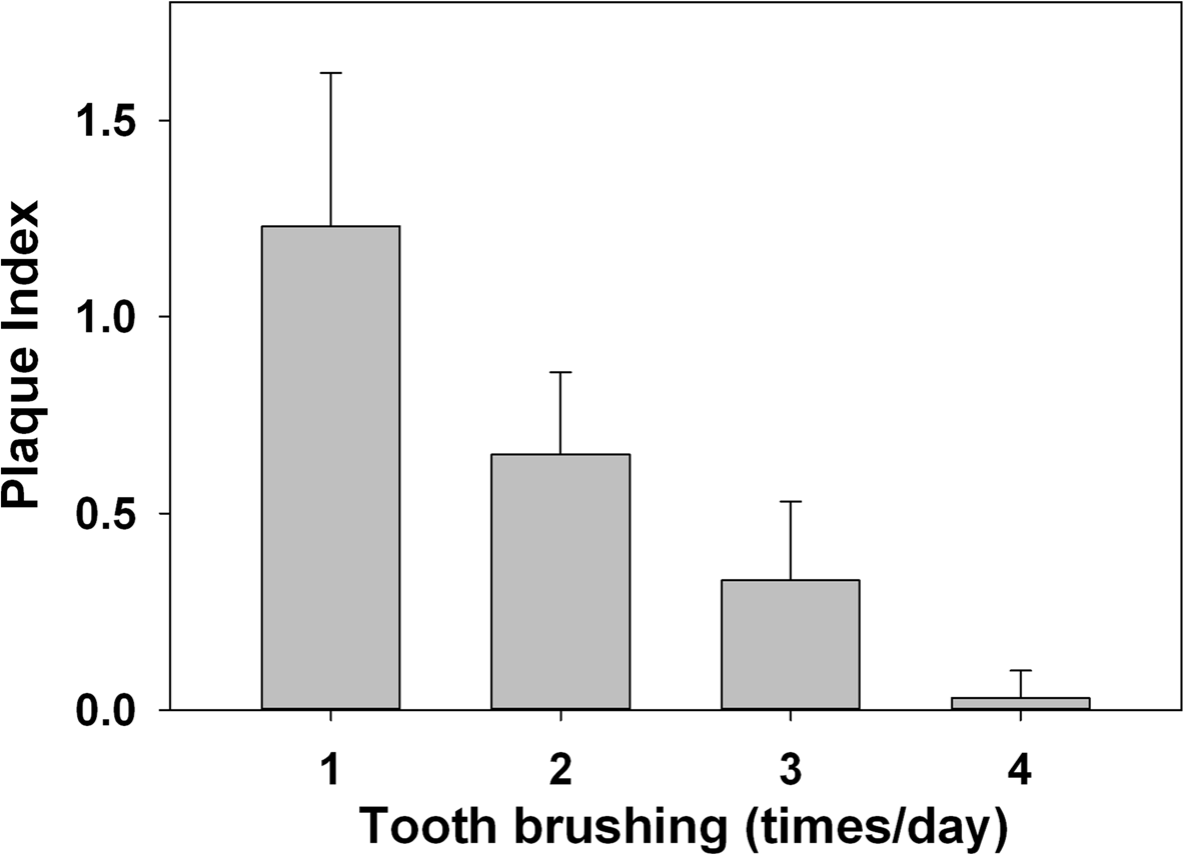
Biofilm formation in patients with different daily tooth brushing frequencies. A significantly association was found between the frequency of daily tooth brushing and the amount of biofilm formation (*P* < 0.001). Note the pronounced gradient showing less biofilm in patients reporting higher frequency of tooth brushing

Male patients (0.9 ± 0.5) had significantly more biofilm than female patients (0.6 ± 0.5) (*P* < 0.001), whereas children (<18 years old) (0.8 ± 0.6) had significantly more biofilm than adults (≥18 years old) (0.3 ± 0.3) (*P* < 0.001) (Fig. 3). Furthermore, patients who were “family-motivated” to orthodontic treatment had more biofilm accumulation (1.1 ± 0.5) than patients who were “self- and family-motivated” (0.7 ± 0.6) and “self-motivated” (0.3 ± 0.3) (*P* < 0.001) (Fig. 4). In addition, strong association was found between the frequency of tooth brushing and the amount of biofilm formation (*P* < 0.001), which showed a negative gradient (Fig. 5).

## Discussion

Biofilm-related side effects during orthodontic treatment are common and may severely impact the quality of treatment outcome as well as patient’s quality of life. Hence, it is important for clinicians to gain insights into where dental biofilm accumulates. Understanding the locations at risk for biofilm formation can aid orthodontists and patients when introducing preventive strategies to minimize the development of WSLs.

The distribution of dental biofilm in non-orthodontic samples has been previously investigated [17]. Molars were found to accumulate more biofilm than anterior teeth, and the mandibular dentition harbored more biofilm than the maxillary dentition [17]. These observations are in contrast with the pattern of biofilm distribution found in our orthodontic patients, where the upper lateral incisors and upper canines generally had more biofilm accumulation than the upper and lower premolars and the maxillary arch accumulated more biofilm than the mandibular arch. The maxillary lateral incisors and canines accumulated the highest level of dental biofilm, which may be because these teeth are located at the corners of the mouth and receive relatively less tooth brushing strokes during daily oral hygiene. Furthermore, the orthodontic hooks and elastics are usually attached to these areas, making it more difficult to clean [18].

The site-specific distribution of biofilm in patients wearing fixed appliances could help to explain, at least in part, the considerable variation in the distribution of WSLs seen between individuals and at different sites within the same mouth [2, 19]. Though most subjects generally used their right hand to brush their teeth, in this study, no significant difference was found in the biofilm formation between the left and right sides, consistently with previous findings [9, 20]. The gingival, distal, and mesial areas, in relation to the brackets, attracted more biofilm than the occlusal areas, which was mostly due to the interference of arch wires and ligating devices on tooth brushing. There is also relatively less self-cleaning from natural chewing in these areas [21].

The pattern of biofilm distribution seen in our orthodontic patients is also consistent with previous observations that WSLs occur 2.5 times more frequently in the maxillary arch than in the mandibular arch, with the most prevalent areas being the maxillary lateral incisors, maxillary canines, molars, and mandibular canines [1, 2, 22].

The plaque index (PI) has been widely used for assessing the level of biofilm formation [15]. However, it was originally designed for normal populations in the absence of fixed appliances. Therefore, in order to obtain more valid and discriminatory PI measurements, we used the modified PI system in the study, which acknowledges the impact of brackets on biofilm distribution and has greater categorical discrimination than the original Silness and Löe index [14, 15].

In our study, self-motivated patients were found to be more cooperative with the clinician’s instructions than self- and family-motivated and family-motivated categories, resulting in a significant difference in biofilm formation among the three categories of patients in the study. This indicates that motivation to undergo orthodontic treatment is important when attempting to predict patient cooperation [23, 24]. A lack of cooperation has a significantly negative effect on the results and duration of treatment, as well as on oral hygiene maintenance.

Our findings suggest that adults and females had less biofilm than children and girls. This is consistent with previous observations that adults and females adhere better to clinician’s instructions and consequently may maintain better oral hygiene [9, 16, 23]. Orthodontists should therefore take age and gender differences into account when delivering oral hygiene measures.

As expected, our study found that the higher the self-reported frequency of tooth brushing, the less the biofilm formation [25, 26]. Despite the inherent limitation of self-reported assessments, this finding emphasizes the need for sufficient tooth brushing in patients with fixed appliances, especially on teeth more at risk of biofilm formation such as the maxillary lateral incisors and maxillary canines.

One of the limitations of the study is the cross-sectional nature, which makes it difficult to make a causal inference. Our sample size was relatively small, and this represents a limitation of the present study. Additionally, some patients were extraction cases, for example premolar extractions. This may have reduced the power of some of our statistical comparisons. Although the differences of biofilm formation among different variables were mostly found statistically significant in the study, the clinical significance may be not. However, the differences of biofilm formation become more pronounced in selected individuals [9, 12]. Furthermore, the differences in wires, loops, ligature, and auxiliary devices used in patients may also influence the generalizability of our results. Future studies with larger sample sizes and homogeneous fixed appliances are needed. Researchers in the future should aim to design more effective and specific methods to improve oral hygiene in patients with fixed appliances, particularly on areas of teeth that accumulate more biofilm.

## Conclusions

Patients wearing fixed orthodontic appliances have the highest biofilm accumulation on the maxillary lateral incisors and maxillary canines, particularly in the gingival area and areas behind arch wires. Less biofilm was observed in female and adult patients and in those who were self-motivated and reported brushing their teeth more often.

## Abbreviations

PI: Plaque index
WSLs: White spot lesions
